# The Medical Society of the State of New York

**Published:** 1903-03

**Authors:** 


A Monthly Review of Medicine and Surgery.

*EDITOR:*

WILLIAM WARREN POTTER, M. D.

All communications, whether of a literary or business nature, books for review and exchanges should be addressed to the editor: 284 FRANKLIN STREET, BUFFALO, N. Y.

VOL. XLII.—LVIII. MARCH, 1903. No. 8.

### The Medical Society of the State of New York.

THE 97th annual meeting of this society, held at Albany, January 27, 28 and 29, 1903, was from every viewpoint a most satisfactory medical gathering. In the number that attended, in the quality of the papers read, in the official reports presented, it was everything to be desired. The president, Dr. Henry Reed Hopkins, of Buffalo, has abundant reason to be satisfied with his own labors as well as with the work of his business committee and other coadjutors, which culminated in such a splendid result.

The president's inaugural address dealt with several items of particular interest, two of which claim attention as of great, if not paramount importance. Dr. Hopkins's suggestion that public health officers be trained in sanitary science and that the instruction shall be of such a nature as to lead up to the degree of Doctor of Public Health, to be conferred by the regents of the University upon examination, is one that appeals to every educator and especially to every physician as being in keeping with the progress of the century. This is a question that ought not to admit of two opinions concerning its rightful propriety.

The other recommendation of the president—namely, that the society consider the propriety of taking steps looking to the proper observance of its centenary which is approaching, was approved. The president was authorised to appoint a committee which should deliberate upon the feasibility of appropriately celebrating the event and report its suggestions at the annual meeting in 1904. The committee is constituted as follows: chairman, D. B. St. John Roosa, of New York; vice-chairmen, A. Jacobi, New York, and A. Vander Veer, Albany; representing 1st district, Daniel Lewis, New York; 2d district,

George Ryerson Fowler, Brooklyn; 3d district, W. G. Macdonald, Albany; 4th district, Charles Stover, Amsterdam; 5th district, Henry L. Elsner, Syracuse; 6th district, Hamilton D. Wey, Elmira; 7th district, William S. Ely, Rochester; 8th district, William Warren Potter, Buffalo.

The duties of this committee are important, and its work should be conducted upon a liberal basis. It will cost some money to celebrate properly the event in question and the society should exhibit a willingness to defray the expenses, which no doubt will be the case. But we shall have occasion to speak upon this subject again, hence will not consider details at present.

The president advocated the state licensing of nurses, spoke of the progress making in the establishment of a state hospital for consumptives, and made appropriate reference to the work of the committee appointed last year to promote a resumption of relations with the American Medical Association.

The committee on hygiene, by its chairman, John L. Heffron, of Syracuse, made an excellent report, dealing in part with the prevention of communicable diseases, and proposing improvement in the tenement house law.

The committee to formulate a plan of reorganisation of the profession, in order to effect affiliation with the American Medical Association, of which Dr. Henry L. Elsner is chairman, made an elaborate report in which it stated that as yet it had been unable to come to an understanding with the other societies interested, but it reported progress and asked to be continued. The committee was empowered to proceed with its work for another year.

The program was full of important material and occupied the society most industriously for six sessions. Two symposiums, one on hematology, and the other on arteriosclerosis, attracted great attention and deep interest. The latter deserves more extended comment than we are able to give in this place, because of the influence it will exert in the etiology, pathology and treatment of this condition.

Dr. Charles A. L. Reed, of Cincinnati, delivered an oration on the life and character of Rudolph Virchow, which is a model of rhetorical and biographical eulogium. We shall print it in a future number of the JOURNAL.

The president's anniversary address was a plea for Progress, Unity and Liberty, the underlying thought of which was fealty to the law as the only safety of citizenship, the responsibility of the physician being greater in this respect because of his education; that unity could only come through such fealty, and, finally, that true progress could only be made through the liberty that unity would ensure. It is printed in full in the JOURNAL for February.

The annual banquet at The Ten Eyck was a social climax to an intellectual feast that had been in progress for two days. The 400 banqueters were joyous in the atmosphere of music, song and speech. The event of the evening was the address of Hon. J. Sloat Fassett, the subject of which was The Empire State and the Twentieth Century. Rarely does a post-prandial effort meet with such a reception and still more rarely have such speeches been as worthy of approval. His lofty thoughts, clothed in finished rhetoric, and accentuated with graceful gesticulations were worthy the man and the occasion. Mr. Fassett deserves the thanks of every doctor who heard him, for his brilliant word-painting of a magnificent subject.

The officers elected were: *President*, A. T. Bristow, of Brooklyn; *Vice-President*, Edward B. Angell, of Rochester; *Secretary*, Frederic C. Curtis, of Albany; *Treasurer*, O. D. Ball, of Albany.

